# Dairy Calves in Uruguay Are Reservoirs of Zoonotic Subtypes of *Cryptosporidium parvum* and Pose a Potential Risk of Surface Water Contamination

**DOI:** 10.3389/fvets.2020.00562

**Published:** 2020-08-21

**Authors:** Rubén Darío Caffarena, Marcelo Vasconcelos Meireles, Leonidas Carrasco-Letelier, Catalina Picasso-Risso, Bruna Nicoleti Santana, Franklin Riet-Correa, Federico Giannitti

**Affiliations:** ^1^Instituto Nacional de Investigación Agropecuaria (INIA), Plataforma de Investigación en Salud Animal, Estación Experimental INIA La Estanzuela, Colonia, Uruguay; ^2^Facultad de Veterinaria, Universidad de la República, Montevideo, Uruguay; ^3^Faculdade de Medicina Veterinária, Universidade Estadual Paulista, Araçatuba, Brazil; ^4^Instituto Nacional de Investigación Agropecuaria (INIA), Programa de Producción y Sustentabilidad Ambiental, Estación Experimental INIA La Estanzuela, Colonia, Uruguay; ^5^Department of Veterinary Population Medicine, College of Veterinary Medicine, University of Minnesota, Saint Paul, MN, United States

**Keywords:** bovine cryptosporidiosis, *Cryptosporidium parvum* zoonotic subtypes, dairy calves, diarrhea, spatial clusters, surface water, Uruguay

## Abstract

*Cryptosporidium parvum*, a major cause of diarrhea in calves, is of concern given its zoonotic potential. Numerous outbreaks of human cryptosporidiosis caused by *C. parvum* genetic subtypes are reported yearly worldwide, with livestock or water being frequently identified sources of infection. Although cryptosporidiosis has been reported from human patients in Uruguay, particularly children, epidemiologic information is scant and the role of cattle as reservoirs of zoonotic subtypes of *C. parvum* has not been explored. In this study, we aimed to (a)-identify *C. parvum* subtypes infecting dairy calves in Uruguay (including potentially zoonotic subtypes), (b)-assess their association with calf diarrhea, (c)-evaluate their spatial clustering, and (d)-assess the distance of infected calves to surface watercourses draining the farmlands and determine whether these watercourses flow into public water treatment plants. Feces of 255 calves that had tested positive for *Cryptosporidium* spp. by antigen ELISA were selected. Samples had been collected from 29 dairy farms in seven Uruguayan departments where dairy farming is concentrated and represented 170 diarrheic and 85 non-diarrheic calves. Selected samples were processed by nested PCRs targeting the 18S rRNA and gp60 genes followed by sequencing to identify *C. parvum* subtypes. Of seven *C. parvum* subtypes detected in 166 calves, five (identified in 143 calves on 28/29 farms) had been identified in humans elsewhere and have zoonotic potential. Subtype IIaA15G2R1 was the most frequent (53.6%; 89/166), followed by IIaA20G1R1 (24.1%; 40/166), IIaA22G1R1 (11.4%; 19/166), IIaA23G1R1 (3.6%; 6/166), IIaA17G2R1 (3%; 5/166), IIaA21G1R1 (2.4%; 4/166), and IIaA16G1R1 (1.8%; 3/166). There were no significant differences in the proportions of diarrheic and non-diarrheic calves infected with any of the *C. parvum* subtypes. Two spatial clusters were detected, one of which overlapped with Uruguay's capital city and its main water treatment plant (Aguas Corrientes), harvesting surface water to supply ~1,700,000 people. Infected calves on all farms were within 20–900 m of a natural surface watercourse draining the farmland, 10 of which flowed into six water treatment plants located 9–108 km downstream. Four watercourses flowed downstream into Aguas Corrientes. Calves are reservoirs of zoonotic *C. parvum* subtypes in Uruguay and pose a public health risk.

## Introduction

Cryptosporidiosis is a global disease caused by protozoa of the genus *Cryptosporidium*. It affects a wide variety of hosts, including humans and ruminants. The predominant species that infect cattle are *C. parvum, C. andersoni, C. bovis*, and *C. ryanae* (1). *C. parvum* is a major cause of diarrhea in neonate calves, which shed large amounts of highly resistant fecal oocysts that contaminate the environment (2). Moreover, it is a zoonotic pathogen and a leading cause of water- and foodborne diarrheal disease in humans (3). Sources of *Cryptosporidium* infection to humans include contaminated surface water (lakes, rivers), municipal drinking water (as oocysts are largely resistant to chlorination), recreational water (swimming pools, water playgrounds), food, and infected livestock (4–6). Of the numerous outbreaks of human cryptosporidiosis reported annually worldwide (7), many have been linked to cattle as sources of *C. parvum* infection (5, 8, 9).

After fecal-oral transmission, *C. parvum* infects the host enterocytes and undergoes a phase of sexual reproduction, during which the recombination of genes takes place, with the consequent generation of different genetic families and subtypes that, depending on epidemiological conditions, can differ between and within geographical regions (10, 11). It is not possible to identify *Cryptosporidium* to the species level or *C. parvum* subtypes with conventional techniques traditionally used to detect cryptosporidia, such as acid-fast or auramine-phenol stains and immunological assays, such as direct immunofluorescence or ELISA. However, genetic analysis of the 18S ribosomal RNA gene allows for *Cryptosporidium* species identification and analysis of the glycoprotein 60 (gp60) locus allows not only for *C. parvum* species confirmation, but also for further identification to the family and subtype levels (12). This molecular approach has been used in epidemiologic studies to assess geographic segregation and interspecies transmission (8, 9), which have led to a better understanding of cryptosporidiosis in animals and humans. For instance, evidence indicates that most infections in young calves are caused by *C. parvum*, primarily from the IIa family, which is known to contain the most frequently zoonotic subtype worldwide IIaA15G2R1 (13–15), regarded as a hyper-transmissible subtype (13). Thus, molecular techniques have aided in the understanding of the epidemiological patterns and transmission chains of cryptosporidiosis and can ultimately help to delineate prevention and control strategies (16).

Exposure to recreational water (35.1%) and direct contact with cattle (14.6%) were the main sources identified in 444 outbreaks of cryptosporidiosis reported in humans in the USA in 2009–2017 (16). Human cryptosporidiosis has been documented in Uruguay (17, 18), although the sources of infection have not been explored. With ~12.2 million head of cattle in 2017–2018, and a total human population of 3.53 million, Uruguay is the country with the highest number of cattle per capita worldwide (19). The area allocated to cattle farms accounts for ~75% of the country's territory (20). Most cattle are raised outdoors in pasture-based farming systems, which causes environmental contamination with feces, exposure of fecal depositions to rainfall, and cattle access to surface natural watercourses. Uruguay's topography is represented by water-rich land and flat plains that sometimes flood. Its humid temperate climate without a dry season (21), annual rainfalls of 700–1,200 mm, along with the dense network of surface natural watercourses (22) averaging 1.4 linear km per km^2^ of area, provide favorable conditions for the transmission of waterborne disease agents.

Considering the reservoir potential of cattle and that *Cryptosporidium* spp. is a frequent cause of diarrhea in dairy calves in Uruguay (23), we wondered whether *Cryptosporidium* species and subtypes infecting cattle could pose a potential risk to public health through either direct contact or surface water contamination. Given the current scenario, in this study, we aimed to (a) identify *Cryptosporidium* species and subtypes infecting dairy calves in Uruguay, including potentially zoonotic *C. parvum* subtypes, (b) assess the association of different *C. parvum* subtypes with calf diarrhea, (c) evaluate their spatial clustering, and (d) assess the distance of infected calves to natural surface watercourses draining the farmlands and determine whether these watercourses flow downstream into public water treatment plants, which may indicate a potential risk to public health.

## Materials and Methods

### Samples and Farms

A total of 255 stool samples from dairy calves stored at −20°C at the Instituto Nacional de Investigación Agropecuaria (INIA) veterinary diagnostic laboratory (Plataforma de Investigación en Salud Animal) were selected for this study. All samples were non-randomized, had been collected for another study between January and November 2016, and had tested positive for *Cryptosporidium* spp. antigen using a commercial antigen capture ELISA kit (Pathasure Enteritis-4; Biovet, St. Hyacinthe, Quebec, Canada) (23), which was an inclusion criterion. Samples represented 170 diarrheic and 85 non-diarrheic dairy calves up to 30 days of age, from 29 commercial dairy farms (farms 1–29) located in seven departments of Uruguay (Colonia, San José, Flores, Soriano, Florida, Canelones and Río Negro). In the original study (23), feces of 552 diarrheic (n: 267, cases) and non-diarrheic (n: 285, controls) neonate dairy calves were sampled. Samples were obtained from commercial farms experiencing spontaneous cases of neonatal diarrhea (convenience sampling). The sample size was calculated using a free online calculator (Epitools, Australia: https://epitools.ausvet.com.au/casecontrolss?page=case-controlSS), considering a power of 80% to detect an association between diarrhea and infection with a given pathogen, a percentage of exposed controls of 5%, and an Odds Ratio (OR) of 2.5 with a 95% confidence level. Of all 552 calves tested by ELISA for *Cryptosporidium* spp. antigen, 265 (48%) resulted positive, 255 of which were available for this study. The sampling protocol was approved by INIA's animal ethics committee for the use of animals in experimentation (CEUA, protocol # 20199).

Additional information was collected from the farms, including herd size (number of milking cows and number of reared calves in 2016), the area (surface in m^2^) of the calf-rearing areas, the type of calf housing in the calf-rearing areas (individual vs. collective or group pens, indoors vs. outdoors), the type of floor in the calf-rearing areas, whether feces were removed from the floor of the calf-rearing areas, and the drinking water sources for the calves (Supplementary Table 1).

### DNA Extraction, PCR Amplification, and Sequencing for *Cryptosporidium* Speciation and Subtyping

DNA was extracted from 150 mg of each of the 255 fecal samples using a commercial kit (Quick DNA Fecal/Soil Microbe Miniprep Kit; Zymo Research, Irvine, CA, USA), following the manufacturer's instructions. A nested PCR protocol targeting the 18S rRNA gene for the detection and speciation of *Cryptosporidium* spp. was performed using the PCR primers 5′-TTCTAGAGCTAATACATGCG-3′ and 5′-CCCATTTCCTTCGAAACAGGA-3′ (~1,319 bp) and the nested PCR primers 5′-GGAAGGGTTGTATTTATTAGATAAAG-3′ and 5′-AAGGAGTAAGGAACAACCTCCA-3 (~834 bp) as previously described (24, 25). Additionally, *C. parvum* speciation and subtyping were performed using a two-step nested PCR protocol targeting a fragment of the gp60 gene using the PCR primers 5′-ATAGTCTCCGCTGTATTC-3′ and 5′-GGAAGGAACGATGTATCT-3′ (~900 bp), and the nested PCR primers 5′-TCCGCTGTATTCTCAGCC-3′ and 5′-GCAGAGGAACCAGCATC-3′ (~860 bp) (26), in 166 (74.8%) of the 18S rRNA PCR-positive samples. Samples were selected for speciation and subtyping ensuring that all 29 farms were represented. Amplification reactions for the 18S rRNA and gp60 genes were performed in a volume of 25 μL containing Platinum® PCR SuperMix (Life Technologies, Carlsbad, CA, USA), 200 nM of each primer and 2 μL of target DNA in both PCR and nested PCRs. Reactions were performed on a CFX96^TM^ Real-Time PCR Detection System (Bio-Rad, Hercules, CA, USA). Samples were denaturated at 94°C for 2 min, followed by 40 cycles of denaturation at 94°C for 30 s, annealing for 30 s. at 55°C (18S rRNA gene) or 50°C (gp60 gene) and extension for 1 min. at 72°C, with a final extension at 72°C for 7 min. *C. parvum* DNA and ultrapure water were used as positive and negative controls, respectively. Amplified fragments were analyzed by GelRed® (Biotium, Fremont, CA, USA) stained gel electrophoresis.

The obtained amplicons for both the 18S rRNA and gp60 genes were purified using a QIAquick Gel Extraction Kit (Qiagen, Santa Clara, CA, USA) and sequenced using the ABI Prism® Dye Terminator Cycling Sequence kit in an ABI 3730XL automatic sequencer (Applied Biosystems, Foster City, CA, USA). DNA sequences were assembled and aligned with CodonCode Aligner version 7.1.2 (CodonCode Corporation, Centerville, MA, USA) and BioEdit Sequence Alignment Editor (27), and compared with homologous sequences available in GenBank using Clustal W (28). *C. parvum* subtypes were identified based on the number of TCA (A), TCG (G), and ACATCA (R) repeats (29).

### Statistical Analyses

Comparisons among proportions of diarrheic and non-diarrheic calves infected with each different *C. parvum* subtype were performed with a one-sample *z-*test of proportion (when *n* ≥ 30) or binomial test (when *n* < 30) for a significance level of *P* < 0.05 using STATA® version 14.0 (StataCorp, College Station, TX, USA).

For spatial cluster detection, multinomial modeling of the spatial scan statistics was performed to assess the relative risk for clusters of each *C. parvum* subtype detected at the farm level, as described previously (30, 31). Briefly, different sized circular windows are placed randomly over the area of study and the likelihood ratio of the cases (each *C. parvum* subtype) clustered within the window is compared with the expected in the remaining areas as generated by 999 Monte Carlo simulations. Cluster detection was implemented in SaTScan™ version 9.4.4 (Martin Kulldorff, Boston, MA, USA).

### Assessment of the Location and Distance Between Infected Calves, Natural Surface Watercourses, and Downstream Surface Water Treatment Plants

The calf-rearing areas of the 29 dairy farms where the infected calves were located were georeferenced and mapped. The elevation and the slope of the farmland, obtained from the digital terrain model of Uruguay (32), were considered to assess the water drainage network for each calf-rearing area. The distance between each calf-rearing area and the nearest surface natural watercourse (streams or rivers) was measured in meters with QGIS software (33), considering the shortest natural water drainage route. Additionally, we assessed whether these natural surface watercourses would flow downstream in the direction of any public water treatment plants harvesting surface water for sanitation and human consumption. The geographic locations of these water plants was obtained from the website of the Uruguayan “Ministerio de Vivienda, Ordenamiento Territorial y Medio Ambiente” (22), and the distance between these points following the path of the watercourses down to the treatment plants was measured in kilometers using QGIS software.

## Results

### *Cryptosporidium* Species and Subtype Identification

Of the 255 samples included in the study, 222 (87.1%) were positive by the nested PCR targeting the 18S rRNA gene, confirming *Cryptosporidium* spp. To conduct *Cryptosporidium* species identification, 60 (27%) of the 222 18S rRNA PCR-positive samples, selected at random and representing all 29 farms, were further sequenced; *C. parvum* was the only species identified in all 60 calves. Based on these results, considering that *C. parvum* is the main species found in calves up to 30 days of age (15), and due to financial constraints, we decided to pursue additional *C. parvum* speciation and subtyping using a nested PCR assay targeting the gp60 gene followed by sequencing. This approach was followed in 166 (74.8%) of the 18S rRNA PCR-positive samples, including the above-mentioned 60 samples identified as *C. parvum* by 18S rRNA amplicon sequencing, and representing all 29 farms. One of seven different *C. parvum* subtypes were identified in all 166 calves (Table 1). Nucleotide sequences generated in this study (18S rRNA and gp60 genes) were deposited in GenBank under accession numbers MT010356 through MT010363.

**Table 1:** Number of <30-day-old dairy calves infected with different *Cryptosporidium parvum* subtypes in seven departments of Uruguay.

**Department**	***C. parvum*** **subtypes**							**Total**
	^*^IIaA15G2R1	^*^IIaA20G1R1	**IIaA22G1R1**	^*^IIaA23G1R1	^*^IIaA17G2R1	**IIaA21G1R1**	^*^IIaA16G1R1	
Colonia	19	0	10	6	0	0	0	35
San José	48	9	1	0	5	4	3	70
Flores	4	1	0	0	0	0	0	5
Soriano	8	0	0	0	0	0	0	8
Florida	8	0	1	0	0	0	0	9
Canelones	1	0	0	0	0	0	0	1
Río Negro	1	30	7	0	0	0	0	38
Total	89	40	19	6	5	4	3	166

* *Subtypes that have been found in humans elsewhere: IIaA15G2R1 (34), IIaA16G1R1 (35, 36), IIaA17G2R1 (35, 37, 38), IIaA20G1R1 (39), and IIaA23G1R1 (40)*.

The most frequent subtype in the 166 calves was IIaA15G2R1 (53.6%; 89/166), followed by IIaA20G1R1 (24.1%; 40/166), IIaA22G1R1 (11.4%; 19/166), IIaA23G1R1 (3.6%; 6/166), IIaA17G2R1 (3%; 5/166), IIaA21G1R1 (2.4%; 4/166), and IIaA16G1R1 (1.8%; 3/166). Subtype IIaA15G2R1 was also the most frequent at the farm level (20/29, 69%), and was identified in 100% of the seven departments. Subtype IIaA20G1R1 was identified in 8/29 (27.6%) farms and 3/7 (42.9%) departments (Río Negro, San José and Flores). Subtype IIaA22G1R1 was identified in 6/29 farms (20.7%) in 3/7 departments (Colonia, Río Negro and Florida). The other four subtypes (IIaA23G1R1, IIaA17G2R1, IIaA21G1R1, and IIaA16G1R1) were only identified in individual farms, one in Colonia and three in San José. The latter department was the only one where all seven different *C. parvum* subtypes were found. In seven of the 29 farms (24.1%) more than one subtype of *C. parvum* was found. The combination of subtypes included IIaA15G2R1—IIaA22G1R1 and IIaA15G2R1—IIaA20G1R1 in three farms each, and IIaA15G2R1—IIaA21G1R1 in a single farm. At least one zoonotic subtype was identified in 28 of the 29 (96.6%) farms.

### *Cryptosporidium parvum* Subtypes and Calf Diarrhea

Of the 166 samples subjected to subtyping, 119 were from diarrheic and 47 from non-diarrheic calves. There were no significant differences in the proportions of diarrheic and non-diarrheic calves infected with any of the *C. parvum* subtypes (Table 2).

**Table 2:** Proportion of *Cryptosporidium parvum* subtypes in non-diarrheic and diarrheic calves, and results of the one-sample test of proportion or binomial test.

***C. parvum* subtype**	**Consistency of feces**						
	**Non-diarrheic**		**Diarrheic**		**Total**		***P*-value**
	***n***	**Proportion, 95%CI**	***n***	**Proportion, 95%CI**	***n***	**Proportion, 95%CI**	
IIaA15G2R1	21	45%, 3.1–58.7	68	57%, 48.1–65.6	89	54%, 46.0–61.0	0.253
IIaA20G1R1	15	32%, 20.4–46.1	25	21%, 14.6–29.2	40	24%, 18.2–31.1	0.203
IIaA22G1R1	2	4%, 1.2–14.2	17	14%, 9.1–21.7	19	11%, 7.5–17.2	0.139
IIaA23G1R1	2	4%, 0.5–14.5	4	3%, 0.9–8.3	6	4%, 1.3–8.0	0.239
IIaA17G2R1	3	6%, 1.3–17.5	2	2%, 0.2–5.9	5	3%, 0.9–7.0	0.166
IIaA21G1R1	3	6%, 1.3–17.5	1	0.8%, 0.02–5.0	4	2%, 0.6–6.0	0.103
IIaA16G1R1	1	2%, 0.05–11.3	2	0.2%, 0.2–6.0	3	2%, 0.3–5.2	0.574
Total	47		119		166		

*CI, confidence interval*.

### *Cryptosporidium parvum* Subtypes and Spatial Analysis

**Figure 1:**
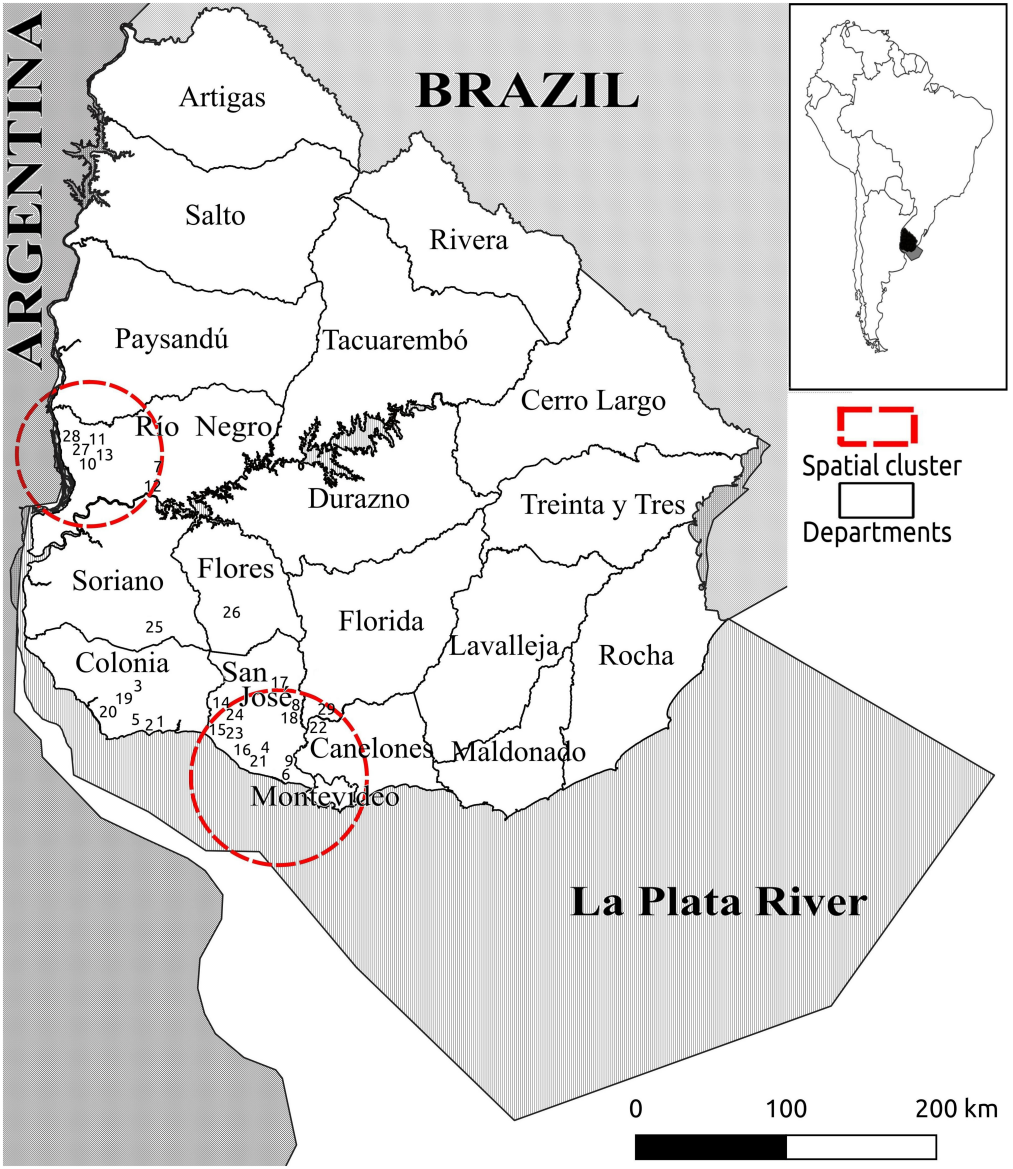
Map of Uruguay (entire country) showing the geographic distribution of 29 dairy farms with *Cryptosporidium parvum*-positive calves. Numbers indicate the farm identification (1–29). Red lines indicate spatial clusters for different *C. parvum* subtypes, as shown in Table 4.

**Figure 2:**
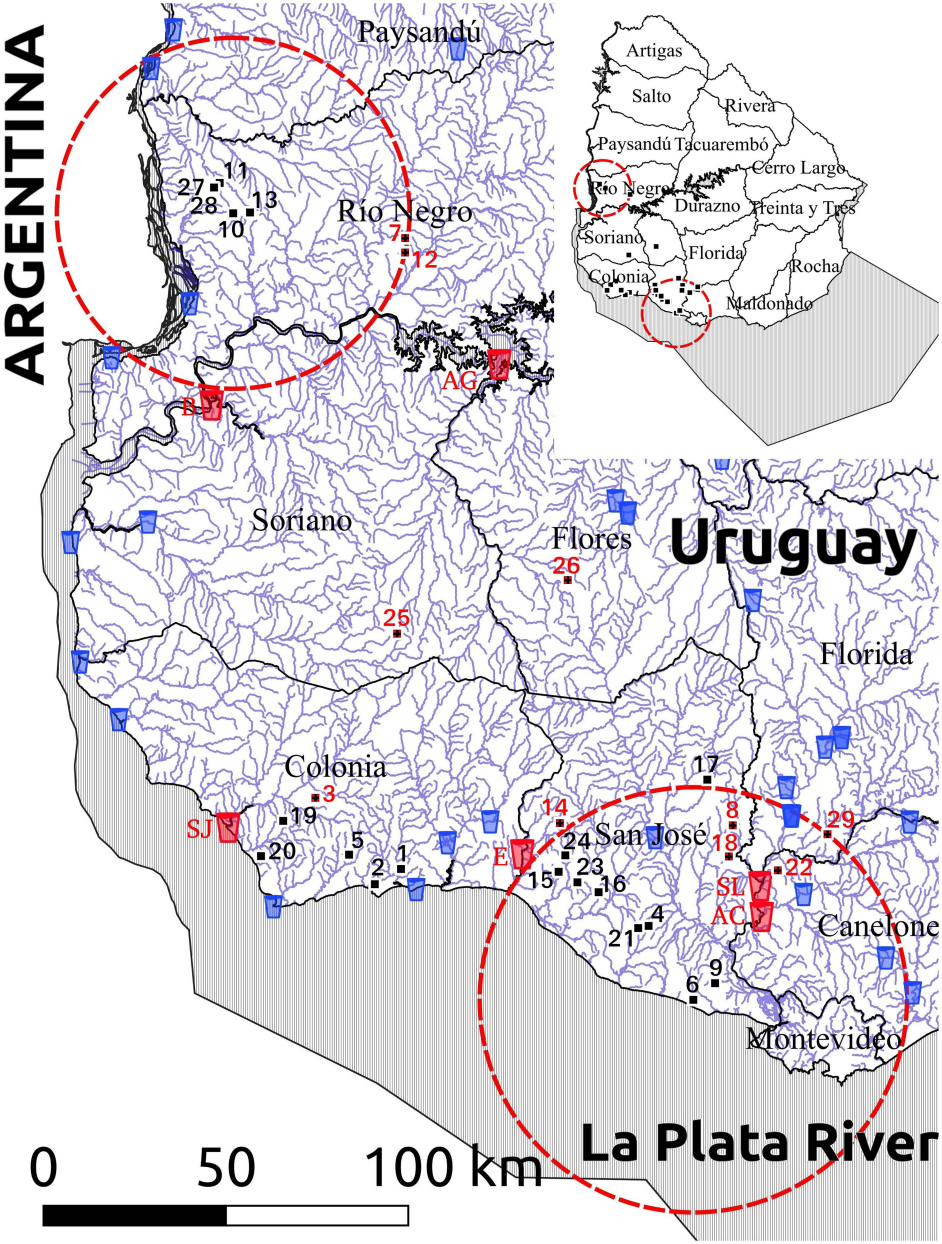
Map of the study area (southwestern Uruguay) showing the geographic distribution of the 29 dairy farms with *Cryptosporidium parvum*-infected calves (numbers), natural surface watercourses (blue lines) and water treatment plants harvesting water for human consumption (glass icons). Ten farms that drain into a watercourse that flows downstream into a water treatment plant are indicated with red numbers, while the six water treatment plants receiving water from these 10 watercourses are highlighted with red glass icons and identified as AC (Aguas Corrientes), AG (Arroyo Grande), B (Bequeló), E (Ecilda), SJ (San Juan), and SL (Santa Lucía). Red lines indicate spatial clusters for different *C. parvum* subtypes, as shown in Table 4.

The geographic locations of the 29 farms are shown in Figures 1, 2, and the outputs of the spatial analysis are summarized in Table 3. Two spatial clusters were identified (*P* < 0.0001). The primary cluster comprised seven farms, located in most of Río Negro, the south of Paysandú, and the north of Soriano (Figures 1, 2). The cluster included a total of 38 infected calves, with the main subtypes being IIaA20G1R1 and IIaA22G1R1 (Table 3). The secondary cluster comprised 12 farms located in the departments of San José, Florida, and Canelones, overlapping with the country's largest metropolitan area and capital city, Montevideo (Figure 2). The total number of infected calves in this cluster was 67, and the main subtypes identified were IIaA15G2R1, IIaA17G2R1, and IIaA21G1R1.

**Table 3:** Outputs of the spatial analysis for *Cryptosporidium parvum* subtype cluster detection in dairy calves.

**Cluster rank**	**Number of dairy herds in each cluster**	**Centroid geolocation**		**Radius (km)**	**Total number of cases**	**Subtype**	**Observed/expected cases for each subtype**	**RR**	**LLR**	***P*-value**
		**South**	**West**							
1	7	32.757967	57.931379	48.21	38	IIaA15G2R1	0.049	0.038	48.821332	<0.0001
						IIaA16G1R1	0	0		
						IIaA17G2R1	0	0		
						IIaA20G1R1	3.28	10.11		
						IIaA21G1R1	0	0		
						IIaA22G1R1	1.61	1.96		
						IIaA23G1R1	0	0		
2	12	34.718798	56.602989	58.42	67	IIaA15G2R1	1.56	2.51	46.881090	<0.0001
						IIaA16G1R1	0	0		
						IIaA17G2R1	2.48	Infinity		
						IIaA20G1R1	0	0		
						IIaA21G1R1	2.48	Infinity		
						IIaA22G1R1	0.26	0.17		
						IIaA23G1R1	0	0		

*RR, relative risk; LLR, log likelihood ratio*.

### Location and Distance Between *Cryptosporidium parvum*-Infected Calves, Natural Watercourses, and Downstream Surface Water Treatment Plants

A map showing the natural surface watercourses of the study area and the location of farms with *C. parvum*-positive calves is depicted in Figure 2. The distance between the calf-rearing areas on these farms and the nearest watercourse (considering the shortest water drainage route based on the altitude and slope of the terrain), as well as the subtypes detected in each farm, are shown in Table 4. The average distance between the calf-rearing areas with *C. parvum*-positive calves (n: 29) and natural surface watercourses was 352 m, with a range of 20–900 m. Ten of these 29 (34.5%) watercourses flowed downstream into six surface water treatment plants located in the departments of Canelones, Flores, Soriano, San José, and Colonia (Figure 2 and Table 4). The average distance between these 10 calf-rearing areas and the respective nearest natural watercourse was 311 m (range: 20–700 m), and the average distance between these points and the closest downstream water treatment plants was 52.95 km (range: 9–108 km). Four of these watercourses, which drained farms in the secondary spatial cluster, flowed downstream into the Santa Lucía River in Canelones, and further down into two water treatment plants (Santa Lucía and Aguas Corrientes) that overlapped with the secondary spatial cluster (Figure 2 and Table 4). The average distance between these four calf-rearing areas and the nearest watercourse was 307.5 m, with a range of 50–550 m; the distances to these two water treatment plants are shown in Table 4. At least one zoonotic subtype of *C. parvum* was identified in 28 of the 29 (96.6%) farms, including 9/10 (90%) farms located upstream from water treatment plants (Table 4).

**Table 4:** Distance of farms with calves infected with different *Cryptosporidium parvum* subtypes from surface natural watercourses and downstream water treatments plants.

**Farm ID**	**Distance of the calf-rearing areas to the nearest draining surface natural watercourse (m)**	**Does the watercourse flow downstream into surface water treatment plants?**	**Surface water treatment plant ID and department**	**Distance from the watercourse to the downstream water treatment plant (km)**	***C. parvum* subtypes and calves**	**Spatial cluster**
1	300	No	–	–	^*^IIaA15G2R1 (7 calves)	None
2	200	No	–	–	IIaA22G1R1 (2 calves), ^*^IIaA15G2R1 (1 calf)	None
3	20	Yes	San Juan, Colonia	30	^*^IIaA23G1R1 (6 calves)	None
4	40	No	–	–	^*^IIaA15G2R1 (3 calves)	Secondary
5	900	No	–	–	IIaA22G1R1 (7 calves), ^*^IIaA15G2R1 (1 calf)	None
6	265	No	–	–	^*^IIaA15G2R1 (8 calves)	Secondary
7	250	Yes	Bequeló, Soriano	108	IIaA22G1R1 (7 calves)	Primary
8	50	Yes	Santa Lucía (SL) and Aguas Corrientes (AC), Canelones	25.5 (SL), 36.5 (AC)	^*^IIaA17G2R1 (5 calves)	Secondary
9	700	No	–	–	^*^IIaA15G2R1 (4 calves)	Secondary
10	600	No	–	–	^*^IIaA20G1R1 (7 calves)	Primary
11	500	No	–	–	^*^IIaA20G1R1 (3 calves)	Primary
12	150	Yes	Bequeló, Soriano	101	^*^IIaA20G1R1 (5 calves)	Primary
13	620	No	–	–	^*^IIaA20G1R1 (4 calves), ^*^IIaA15G2R1 (1 calf)	Primary
14	300	Yes	Ecilda, San José	20	^*^IIaA16G1R1 (3 calves)	None
15	90	No	–	–	^*^IIaA15G2R1 (4 calves)	Secondary
16	365	No	–	–	^*^IIaA15G2R1 (10 calves)	Secondary
17	340	No	–	–	^*^IIaA20G1R1 (9 calves), ^*^IIaA15G2R1 (1 calf)	None
18	450	Yes	SL and AC, Canelones	15 (SL), 26 (AC)	^*^IIaA15G2R1 (5 calves)	Secondary
19	200	No	–	–	^*^IIaA15G2R1 (5 calves)	None
20	400	No	–	–	^*^IIaA15G2R1 (3 calves)	None
21	860	No	–	–	IIaA21G1R1 (4 calves), ^*^IIaA15G2R1 (3 calves)	Secondary
22	550	Yes	SL and AC, Canelones	9 (SL), 20 (AC)	^*^IIaA15G2R1 (1 calf)	Secondary
23	50	No	–	–	^*^IIaA15G2R1 (6 calves)	Secondary
24	430	No	–	–	^*^IIaA15G2R1 (5 calves)	Secondary
25	460	Yes	Arroyo Grande, Flores	97	^*^IIaA15G2R1 (8 calves)	None
26	700	Yes	Arroyo Grande, Flores	94	^*^IIaA15G2R1 (4 calves), ^*^IIaA20G1R1 (1 calf)	None
27	120	No	–	–	^*^IIaA20G1R1 (3 calves)	Primary
28	120	No	–	–	^*^IIaA20G1R1 (8 calves)	Primary
29	180	Yes	SL and AC, Canelones	30 (SL), 41 (AC)	^*^IIaA15G2R1 (8 calves), IIaA22G1R1 (1 calf)	Secondary

* *Subtypes that have been found in humans elsewhere: IIaA15G2R1 (34), IIaA16G1R1 (35, 36), IIaA17G2R1 (35, 37, 38), IIaA20G1R1 (39), and IIaA23G1R1 (40)*.

### Additional Information of the Farms and Calf-Rearing Areas

In the year of sampling (2016) the farms included in the study had herd sizes that ranged from 70 to 1,260 milking cows and raised between 52 and 1,342 calves in calf-rearing areas ranging from 179 to 7,500 m^2^. In 24 of the 29 farms (82.8%) calves were raised outdoors on dirt floor either in collective pens (n: 13 farms) or individual housing systems (n: 11 farms), while in 4/29 farms (13.8%) calves were raised indoors in individual (n: 2 farms) or collective (n: 2 farms) housing systems with cement (n: 3 farms) or wood floor (n: 1 farm). In all 24 farms raising calves outdoors on dirt floor, calf feces were left on the floor and were not routinely removed from the calf-rearing areas. In the 4 farms raising calves indoors, the cement or wood floor was routinely hosed down, but these facilities did not have a sanitary drainage system for the resulting liquid waste, that overflowed to the adjacent farmland. In all 29 farms (100%) the drinking water source for the calves was untreated underground water. The individual information for each farm and calf-rearing area is summarized in Supplementary Table 1.

## Discussion

In Uruguay, information on *Cryptosporidium* spp. is scarce, dating since 1986, when the parasite was first detected (18). *Cryptosporidium* spp. was identified as a cause of diarrhea in children in 6.15 and 8.9% of the studied population in two independent studies (17, 18), and later considered an emerging disease in this country (41). More recently, *Cryptosporidium* spp. was recognized as a causative agent of neonatal diarrhea in dairy calves (23), and *C. parvum* was identified in shallow watercourses in industrialized areas of the country (42), the latter being the only description of *C. parvum* species confirmation in Uruguay. However, to the best of our knowledge, there are no descriptions of zoonotic cryptosporidiosis in the country. Considering this background information, we wondered whether calves were reservoirs of zoonotic subtypes of *C. parvum* and could pose a potential risk for public health either through direct contact or surface water contamination.

*Cryptosporidium* spp. is a major cause of diarrhea in dairy calves in Uruguay. In a case-control study by our group, *Cryptosporidium* spp. antigen was identified by capture ELISA in feces of 189/271 (69.7%) diarrheic and 79/285 (27.7%) non-diarrheic calves from 100% of 30 dairy farms in seven departments where dairy farming is concentrated. Infected calves were six times more likely to be diarrheic than non-infected ones (23). This indicates that cryptosporidiosis is widespread in calves, although the eventual role of cattle as reservoir of zoonotic *Cryptosporidium* strains had not been explored, as the molecular identification of *Cryptosporidium* species and subtypes infecting these calves was not pursued. In this follow-up study, using mostly the same sample set, we showed the wide distribution of *C. parvum* at the calf and dairy farm levels, even with a limited sample size that is unlikely to be representative of the dairy cattle population of the country. The predominance of *C. parvum* is somewhat expected and consistent with the age of the studied calves, as this is the most frequent species in neonate animals (15). In all the farms and departments included in the study, at least one subtype of *C. parvum* was identified, as in other studies carried out in Europe (43–46), Australia (47), Argentina (11, 48, 49), New Zealand (50), Brazil (51), Chile (52), and Colombia (53). However, the results differ from those observed in Sweden (54), China (55, 56), Canada (57), and Ethiopia (58), where *C. bovis* was the most frequently reported species in cattle of this age range.

The analysis of the sequence of the gp60 gene allowed us to identify seven different *C. parvum* subtypes, all within the family IIa, which is widely recognized for its zoonotic potential (59). The subtypes of the IIa family differ from each other in the number of trinucleotides encoding the amino acid serine, as well as in the number of copies of the ACATCA sequence at the end of the sequence. As observed in other studies from South America, including Argentina (11, 48), Brazil (51), Colombia (53), and Chile (60), all detected subtypes in our study also had only one copy of the ACATCA sequence (R1), with variable copy numbers of the TCA trinucleotide (A15, A16, A17, A20, A21, A22, and A23). Unlike studies from neighboring Argentina (11, 48), we identified two subtypes with two copies of the trinucleotide TCG (G), including subtype IIaA15G2R1 that has also been identified in Brazil, Chile, and Colombia (53, 60, 61), as well as subtype IIaA17G2R1 (35, 37, 38) that so far —of the south American countries—had only been identified in Chile (60) and Brazil (62). In this sense, our study broadens the current knowledge on *C. parvum* subtypes infecting cattle in this subcontinent.

Of the seven subtypes identified in our study, five had been found in humans elsewhere (34–40), and are considered zoonotic. At least one of these five zoonotic subtypes was detected in 28/29 (96.6%) of the farms in all departments in our study, indicating a widespread distribution. As described by other studies (13, 14, 43, 44, 63, 64), the most frequent subtype in our study was IIaA15G2R1. In South America, this subtype has been reported in cattle in Brazil (51), Chile (60), and Colombia (53) but not in Argentina (11, 48, 49). The second and third most frequent subtypes identified in our study (IIaA20G1R1 and IIaA22G1R1) are the most frequent ones found in Argentina (11, 48). Subtype IIaA17G2R1 found in our study was reported in cattle in Australia (38, 65), Italy (63), Germany (43), Brazil (62), and Chile (60), as well as in humans in the USA (37), Malaysia (35), and Australia (38). Interestingly, this subtype was involved in an outbreak of diarrhea in a summer camp in the USA, where there was contact between humans and infected calves, with the concurrent identification of this subtype in samples of both species (5). In Brazil, this subtype was found in the feces of calves, as well as in water from dairy farms (62), suggesting water contamination and waterborne transmission. To the best of our knowledge, there is no published information on the identification of *Cryptosporidium* species or subtypes infecting people or any animal species in Uruguay; thus, our study provides novel information that could be used to frame future studies on diagnostics, molecular epidemiology, and risk assessments.

Although we have previously documented that *Cryptosporidium* spp. infection in dairy calves in Uruguay was statistically associated with neonatal diarrhea (23), none of the subtypes found in this study was statistically associated with this clinical manifestation, indicating that diarrhea is associated with *C. parvum* infection regardless of the subtype. The lack of association between some of these *C. parvum* subtypes in neonate calves under similar rearing conditions has also been documented by other authors (11, 48, 53). Additional studies are necessary to determine the eventual implication of different subtypes in terms of their pathogenicity and elucidate the epidemiology of bovine cryptosporidiosis.

Despite the wide geographic distribution and subtype diversity found in our study, two spatial clusters were detected. In the primary cluster, there was a greater risk for farms to have subtypes IIaA20G1R1 and IIaA22G1R1. In the secondary cluster there was increased risk for subtypes IIaA15G2R1, IIaA17G2R1, and IIaA21G1R1. Geographic differences in *Cryptosporidium* species and subtypes have long been described (10, 34, 66). In our study, one of the clusters was located to the north and the other to the south of the Río Negro. This river could have implications as a natural geographic barrier, which would help to explain these differences, also considering that cattle movement across this river is controlled by a sanitary barrier by the Ministry of Livestock, Agriculture, and Fisheries. Additionally, the cluster in the north, adjacent to Argentina, contained the same subtypes that are more frequent in that country. The cluster located in the south presented the most frequent subtype in cases of zoonosis worldwide (IIaA15G2R1), as well as one involved in waterborne outbreaks of cryptosporidiosis (IIaA17G2R1) (5). As suggested by other authors, the origin of the animals, sources of infection, and management practices probably determine that in some areas there are greater frequencies of certain subtypes (10, 11). Interestingly, one of the spatial clusters identified in our study overlapped with Uruguay's most populated metropolitan area, which includes the capital city, Montevideo. The identification of the geographic distribution and spatial clustering of the subtypes of *C. parvum* facilitate the identification of risk areas for both animals and humans.

It should be considered that cattle movements either within Uruguay or internationally, could eventually determine geographic shifting of different *C. parvum* subtypes over time. In Uruguay, ~16% of dairy farmers send female dairy calves from the farms where they are born, to be custom raised in distant farms referred to as dairy rearing farms (DRFs) that gather tens of thousands of calves owned by many different farmers (67). Once heifers reach puberty, they are bred in the DRFs and sent back to their farms of origin before calving. In turn, neonate male dairy calves are usually sold and transported to beef farms within the country where they are reared for meat. This indicates that *C. parvum* subtypes detected in spatial clusters in this study could potentially spread to other geographic regions of the country.

Similarly, the international trade of livestock represents a potential way of transboundary dissemination of *C. parvum*, and other pathogens. From 2008 to 2016 Uruguay exported over 1.5 million live cattle head to Turkey (53%), Egypt (15%), China (14%), Brazil (5%), Lebanon (3%), and other destinations (9%) (68). As we will discuss in the following paragraphs, many of the zoonotic *C. parvum* subtypes identified in our study have either a limited occurrence or have not been identified in livestock species in countries importing cattle from Uruguay; thus trading provides a chance for transcontinental spread of these subtypes.

The first study on molecular subtyping of *C. parvum* in Turkey, the main country importing cattle from Uruguay, was published in 2012, and analyzed 13 bovine strains. Subtype IIaA15G2R1, the most frequent subtype found in our study, was identified in 10 animals, while subtype IIaA16G3R1 was identified in 2 animals and subtype IIdA15G1 was found in the remainder (69); these two subtypes were not identified in our study. Later, in 2016, a broader study that identified *C. parvum* in 27 dairy calves and 9 goat kids in Turkey revealed subtypes IIaA13G2R1 (20/23), IIdA18G1 (2/23), and IIdA20G1b (1/23) in cattle, and subtypes IIaA13G2R1 (3/8), IIaA15G1R1 (2/8), IIdA22G1 (2/8), and IIdA18G1 (1/8) in goat kids (70). None of these subtypes were identified in our study. Another Turkish study from 2017, described *C. parvum* in 73 of 112 diarrheic goat kids from 12 goat farms (71). Sequence analysis of the gp60 locus could be achieved in 67 cases, and revealed subtypes IIaA14G1R1 and IIaA15G1R1 in 25 goat kids each, IIdA18G1 (n: 9), and IIdA17G1 (n: 8). None of these subtypes were identified in our study. A more recent and even larger study from this country assessed 415 fecal specimens from diarrheic calves (n: 333), lambs (n: 67), and goat kids (n: 15), and identified *C. parvum* in 90 calves, 13 lambs, and 2 goats kids (72). Of the 11 subtypes detected (IIaA11G2R1, IIaA11G3R1, IIaA12G3R1, IIaA13G2R1, IIaA13G4R1, IIaA14G1R1, IIaA14G3R1, IIaA15G2R1, IIdA16G1, IIdA18G1, IIdA22G1) in 82 cases (70 calves, 10 lambs and 2 goat kids), only one (IIaA15G2R1, identified in 4 calves and 3 lambs) was found in our study. Another recent study from Turkey identified *C. parvum* in 138 of 550 calves and heifers (73). Gp60 gene sequence analysis reveled only two subtypes (IIaA13G2R1, IIaA14G1R1) in all 138 samples, none of which were identified in our study. Altogether, this indicates that IIaA15G2R1, the most common subtype identified in Uruguay and regarded as an hyper-transmissible zoonotic subtype, is uncommon in livestock in Turkey (so far only identified in 14 cattle and 3 lambs), and that all other subtypes found in cattle in Uruguay have not been detected in livestock in Turkey, so livestock trading could represents a risk of introduction of these subtypes in the country.

A study on molecular epidemiology of *Cryptosporidium* spp. in 804 livestock (cattle and buffalo) and 165 humans in Egypt sampled in April-June 2011 (74), revealed an overall prevalence of *Cryptosporidium* spp. of 32.3% in livestock (260 animals), and 49.1% in humans (81 cases). *C. parvum* was identified in 142 livestock and 32 humans. All *C. parvum*-positive samples for which a nested gp60 PCR product was obtained were sequenced (n: 120); subtype family IId (which was not identified in our study) was significantly more frequent than subtype family IIa. All *C. parvum* of subtype family IIa detected from cattle (22.5%) and buffalo (11.4%) belonged to the IIaA15G1R1 subtype (which was not identified in our study), while human infections with subtype family IIa (50%) were found to be caused by subtypes IIaA15G1R1 (n: 2, subtype not identified in our study) and IIaA15G2R1 (n: 5, subtype most frequently identified in our study). Another study from Egypt evaluated the prevalence and molecular characteristics of *Cryptosporidium* spp. in dairy cattle in four Nile River delta provinces (75). *Cryptosporidium* spp. were identified in 13.6% of 1,974 fecal specimens obtained from 12 farms between December 2009 and November 2011. Successful amplification and sequencing of the gp60 locus for *C. parvum* subtyping was possible in 37 specimens, 27 were identified as IIaA15G1R1, 9 as IIdA20G1, and 1 as IIaA14G1R1r1b. None of these subtypes were identified in Uruguay. Another study on prevalence and genotyping of *Cryptosporidium* spp. in farm animals in Egypt that evaluated 466 samples from buffalo, 1,697 from cattle and 120 from sheep, identified *C. parvum* in 2 buffalo, 23 cattle and 0 sheep, the subtypes involved being IIdA20G1 and IIaA15G1R1 (76), none of which were identified in our study. Lastly, another study on *Cryptosporidium* genotypes and subtypes in dairy calves in Egypt identified *C. parvum* in 24 from 96 sampled calves, 23 were subtyped as IIdA20G1 (not identified in our study), and only 1 as IIaA15G2R1 (77), which was the most frequent subtype identified in our study. In summary, the most frequent subtype found in Uruguay (IIaA15G2R1), which has been regarded as an hyper-transmissible subtype, has a limited occurrence in Egypt, and to the best of our knowledge, it has so far only been identified in one dairy calf (77) and 5 human patients (74) in this country. In this context, livestock exports from Uruguay could represent a risk of introduction of *C. parvum* subtypes that are either infrequently identified or have not been identified in livestock and people in Egypt.

In China, there is a high diversity of *Cryptosporidium* spp. and subtypes, and the dominant *C. parvum* subtypes detected in this country are rarely detected in other countries. Domestic ruminants (calves, lambs, goat kids) are mostly infected with non-pathogenic *Cryptosporidium* spp., such as *C. bovis* (calves) or *C. xiaoi* (lambs and goat kids). *C. parvum* started to appear in dairy calves as a consequence of concentrated animal feeding operations. Subtyping of *C. parvum* in 9 studies involving dairy calves in 8 geographic areas of China published between 2011 and 2017 identified the exclusive occurrence of IId subtypes, mostly IIdA15G1 and IdA19G1 (78). The few IIa subtypes identified in cattle in China include IIaA15G2R1 (n: 8), IIaA16G2R1 (n: 2), IIaA14G1R1 (n: 1), IIaA14G2R1 (n: 1), and IIaA16G3R1 (n: 1), which were geographically restricted to the Qinghai province (79). Of these subtypes rarely detected in Chinese cattle, only the hyper-transmissible subtype IIaA15G2R1 was identified in Uruguay. *C. parvum* subtypes have also been identified in other domestic ruminants in China, including yak, sheep, and goats. One study identified the exclusive occurrence of a few IIa subtypes in yak, including IIaA15G2R1 (n: 8), IIaA16G2R1 (n: 2), IIaA14G1R1 (n: 1), IIaA14G2R1 (n: 1) and IIaA16G3R1 (n: 1) (80). The IIa subtypes identified in sheep include IIaA15G2R1 and IIaA17G2R1 (81), and those identified in goats include IIaA14G2R1, IIaA15G1R1, IIaA15G2R1 and IIaA17G2R1 (82). Of these seven IIa subtypes identified in yak, sheep, and goats in China, only subtypes IIaA15G2R1 and IIaA17G2R1 were identified in calves in Uruguay. In short, cattle imports from Uruguay could potentially determine the introduction of IIa subtypes that have not been detected or have a limited occurrence in livestock in most Chinese provinces.

Because *Cryptosporidium* is one of the most important waterborne parasites worldwide (7), and given the risk for contamination of aquatic environments by infected animals (57, 83), we assessed the distance between the calf-rearing areas in the studied farms and the closest natural surface watercourses. This was performed by considering the shortest natural drainage route of the farmlands, as well as whether these watercourses would flow downstream into public water treatment plants harvesting surface water for human consumption. This approach revealed some interesting observations. For instance, we found that watercourses draining four farms in the secondary spatial cluster flowed downstream into the Santa Lucía river and further down into Aguas Corrientes water treatment plant, which also overlapped with the secondary spatial cluster. Aguas Corrientes is the country's main water treatment plant, supplying drinking water to ~1.7 million people in the largest metropolitan area in the departments of Montevideo and Canelones (84). The calf-rearing areas in these four farms, lodging 19 calves infected with the zoonotic subtypes IIaA17G2R1 and IIaA15G2R1, were 50, 180, 450, and 550 m away from their respective closest surface watercourses. The calf-rearing area that was closest (20 m) to a natural watercourse was in farm three and lodged six calves infected with the zoonotic subtype IIaA23G1R1. This watercourse flows down into the San Juan water treatment plant in the department of Colonia. Additionally, it should be stated that all the watercourses draining farmlands in this study eventually flow downstream into the Río Negro, Río Uruguay and/or Río de La Plata, all of which line the coast of recreational freshwater beaches in the departments of Río Negro, Soriano, Colonia, San José, Montevideo, and Canelones.

Studies that assessed the presence and concentration of *Cryptosporidium* oocysts in surface watercourses located upstream and downstream from cattle farms found higher proportions and concentrations of oocysts in downstream samples, suggesting that cattle represent a source of surface water contamination with this parasite (57, 85). In one of these studies, the natural watercourses were located within 500 m of the cattle housing facilities (57). Based on this information, it is reasonable to speculate that water draining from the calf-rearing areas in our study (particularly those housing calves outdoors on dirt floor where feces were not removed from the calf-rearing areas) could eventually act as a vehicle of *C. parvum* oocysts to the respective natural watercourses, i.e., after heavy rainfalls leading to surface runoff or floods. Taken together, our results indicate that water contamination by oocysts of zoonotic subtypes of *C. parvum* shed by cattle in Uruguay is likely and could represent a potential risk to public health if people are exposed to natural watercourses or if water used for drinking, recreation, or crop/produce irrigation is not sanitized properly.

Flooding events are frequent in Uruguay. In 2015 and 2016 the El Niño phenomenon caused extremely unstable climatic conditions in the region, causing rivers to swell and overflow their banks. In 2016, the same year of the sampling in our study, the accumulated rainfall in the country was 1,268 mm, 207 mm (19.5%) higher than the average annual accumulated rainfall in the previous decade (2006–2015), which was of 1,061 mm (86). In April of 2016, heavy rains and a tornado caused floods and thousands of flood victims in the entire country, according to Uruguay's national emergency system (87). Such extreme climatic conditions facilitate the environmental contamination with waterborne disease agents.

Additionally, surface water in Uruguay is used by farmers to irrigate crops and produce, which could also represent a possible source of transmission of *C. parvum* to humans through contaminated soil and vegetables (88). Another potential way of exposure of humans to untreated surface freshwater is through recreation. Activities such as swimming, sailing, kayaking, canoeing, fishing, waterskiing, windsurfing, and kiteboarding are commonly practiced in Uruguay by inhabitants and tourists alike, which in 2016 Uruguay accounted for over 3.3 million tourists (89). Furthermore, agritourism is expanding in the country, and some farms offer hands-on experiences with livestock, such as milking cows or herding cattle (90). It should be noted that human outbreaks of cryptosporidiosis with proven bovine-to-human *C. parvum* transmission have been documented in recreational spring pasture events in Sweden (8).

Water contamination probably perpetuates the transmission cycle of cryptosporidiosis in cattle in Uruguay. In all farms included in this study, calves were administered untreated underground water (Supplementary Table 1). In a previous study we found group A rotavirus (RVA) RNA as well as viable and infective RVA particles in drinking water sources administered to neonate dairy calves in Uruguayan farms (91). This indicates that water likely acts as a vehicle in the transmission of causative agents of neonatal calf diarrhea that are less resistant in the environment than *Cryptosporidium* spp. oocysts. Unfortunately, we have not been able to validate laboratory tests to efficiently identify *Cryptosporidium* spp. in water samples.

In Uruguay, 90% of treated water is obtained from superficial sources and undergoes conventional treatment, a physicochemical process consisting of 6 phases: A- pre-treatment, B- coagulation with aluminum sulfate, C- flocculation, D- sedimentation (or flotation), E- filtration, and F- disinfection (chlorination) (92). *Cryptosporidium* spp. oocysts can be physically removed from water supplies by conventional particle separation processes including chemical coagulation-flocculation, sedimentation, and granular media filtration (93). Although conventional water treatment can be effective in reducing *Cryptosporidium* spp. oocyst loads, the effectiveness depends on the initial oocyst concentration in the source water. Additional special physical and chemical processes such as pressure-driven membrane microfiltration or ultrafiltration, or special disinfection procedures such as treatment with ozone or ultraviolet light irradiation, may be required to inactivate *Cryptosporidium* spp. oocysts, as the parasite is highly resistant to chlorination (even at very high doses after prolonged contact time) (93). To the best of our knowledge, such procedures are not available in Uruguayan water treatment plants and drinking water in the country is not specifically screened for *Cryptosporidium* spp. before, during or after the potabilization process. Major waterborne outbreaks of cryptosporidiosis have been linked to evidence of suboptimal water treatment in other countries (6).

## Conclusion

*C. parvum* infection is widespread in dairy calves in Uruguay, and calves are reservoirs of zoonotic *C. parvum* subtypes, the most frequent being IIaA15G2R1 and IIaA20G1R1. Spatial clustering of zoonotic *C. parvum* subtypes in cattle overlapping with highly populated metropolitan areas and natural surface watercourses that flow downstream into public water treatment plants, including the country's main water plant harvesting surface water for human consumption, raises a concern for potential zoonotic waterborne transmission.

## Data Availability Statement

The datasets presented in this study can be found in online repositories. The names of the repository/repositories and accession number(s) can be found in the article/Supplementary Material.

## Ethics Statement

The animal sampling protocol was approved by INIA's animal ethics committee for the use of animals in experimentation (CEUA, protocol # 20199). Verbal informed consent for participation was obtained from the owners of the calves/farms.

## Author Contributions

FG, FR-C, and RC conceived the study. RC, MM, and BS performed molecular testing and DNA sequence analyses. RC and LC-L built the maps and analyzed data. CP-R analyzed data. RC and FG wrote the first draft and final version of the manuscript. MM, LC-L, CP-R, and FR-C wrote parts and/or edited the manuscript. All authors read and approved the content of the manuscript.

## Conflict of Interest

The authors declare that the research was conducted in the absence of any commercial or financial relationships that could be construed as a potential conflict of interest.
