# Corrigendum: Dairy Calves in Uruguay Are Reservoirs of Zoonotic Subtypes of *Cryptosporidium parvum* and Pose a Potential Risk of Surface Water Contamination

**DOI:** 10.3389/fvets.2020.604196

**Published:** 2020-10-29

**Authors:** Rubén Darío Caffarena, Marcelo Vasconcelos Meireles, Leonidas Carrasco-Letelier, Catalina Picasso-Risso, Bruna Nicoleti Santana, Franklin Riet-Correa, Federico Giannitti

**Affiliations:** ^1^Instituto Nacional de Investigación Agropecuaria (INIA), Plataforma de Investigación en Salud Animal, Estación Experimental INIA La Estanzuela, Colonia, Uruguay; ^2^Facultad de Veterinaria, Universidad de la República, Montevideo, Uruguay; ^3^Faculdade de Medicina Veterinária, Universidade Estadual Paulista, Araçatuba, Brazil; ^4^Instituto Nacional de Investigación Agropecuaria (INIA), Programa de Producción y Sustentabilidad Ambiental, Estación Experimental INIA La Estanzuela, Colonia, Uruguay; ^5^Department of Veterinary Population Medicine, College of Veterinary Medicine, University of Minnesota, Saint Paul, MN, United States

**Keywords:** bovine cryptosporidiosis, *Cryptosporidium parvum* zoonotic subtypes, dairy calves, diarrhea, spatial clusters, surface water, Uruguay

In the original article, there was an error in the acknowledgments section. The names of three persons and a staff group were omitted.

A correction has been made to the acknowledgments:

We also thank María Laura Casaux, Carlos Schild, Martín Fraga, and the staff from the Plataforma de Investigación en Salud Animal of INIA for their technical assistance.

