# Supplementary material for: Dairy Calves in Uruguay Are Reservoirs of Zoonotic Subtypes of Cryptosporidium parvum and Pose a Potential Risk of Surface Water Contamination
Source: Front Vet Sci. 2020 Aug 21;7:562. doi: 10.3389/fvets.2020.00562 (PMC7473207; doi:10.3389/fvets.2020.00562)
Supplement: Supplementary file 1 [file Table_1.DOCX]

**Supplementary Table.** Additional information of the farms and calf-rearing areas included in the study

| **Farm ID** | **Number of milking cows in 2016** | **Number of calves reared in 2016** | **Area (surface) of the calf-rearing area (m^2^)** | **Type of calf housing (individual vs collective, indoors vs outdoors)** | **Type of floor in the calf-rearing area** | **Were feces removed from the calf-rearing area?** | **What is the drinking water sources for the calves?** |
| --- | --- | --- | --- | --- | --- | --- | --- |
|  |  |  |  |  |  |  |  |
| 1 | 332 | 168 | 656 | Collective, Outdoors | Dirt | No | UUW |
| 2 | 390 | ND | 640 | Collective, Outdoors | Dirt | No | UUW |
| 3 | ND | ND | 225 | Collective, Outdoors | Dirt | No | UUW |
| 4 | 420 | ND | 942 | Collective, Outdoors | Dirt | No | UUW |
| 5 | 390 | 143 | 416 | Collective, Outdoors | Dirt | No | UUW |
| 6 | 435 | ND | 657 | Collective, Outdoors | Dirt | No | UUW |
| 7 | 250 | 237 | 650 | Individual, Outdoors | Dirt | No | UUW |
| 8 | 500 | 258 | 1138 | Individual, Outdoors | Dirt | No | UUW |
| 9 | 280 | 52 | 248 | Individual, Indoors | Cement | Yes, feces were hosed down to adjacent farmland | UUW |
| 10 | ND | ND | 2007 | Collective, Outdoors | Dirt | No | UUW |
| 11 | 130 | 115 | 507 | Collective, Indoors | Cement | Yes, feces were hosed down to adjacent farmland | UUW |
| 12 | 130 | 76 | ND | Collective, Outdoors | Dirt | No | UUW |
| 13 | 111 | 110 | 360 | Individual, Outdoors | Dirt | No | UUW |
| 14 | 400 | 187 | 1040 | Collective, Outdoors | Dirt | No | UUW |
| 15 | 563 | 281 | 554 | Collective, Indoors | Cement | Yes, feces were hosed down to adjacent farmland | UUW |
| 16 | ND | ND | 1376 | Individual, Outdoors | Dirt | No | UUW |
| 17 | 270 | 145 | 1100 | Individual, Outdoors | Dirt | No | UUW |
| 18 | 230 | 110 | 770 | Individual, Outdoors | Dirt | No | UUW |
| 19 | 140 | ND | 179 | Individual, Indoors | Wood | Yes, feces were hosed down to adjacent farmland | UUW |
| 20 | ND | ND | ND | Individual, Outdoors | Dirt | No | UUW |
| 21 | 1100 | 420 | 5145 | Individual, Outdoors | Dirt | No | UUW |
| 22 | 140 | 62 | 351 | Collective, Outdoors | Dirt | No | UUW |
| 23 | 222 | 216 | 752 | Collective, Outdoors | Dirt | No | UUW |
| 24 | 250 | 276 | 528 | Individual, Outdoors | Dirt | No | UUW |
| 25 | 1260 | 1342 | 4726 | Individual, Outdoors | Dirt | No | UUW |
| 26 | 512 | 286 | 7500 | Individual, Outdoors | Dirt | No | UUW |
| 27 | 70 | 100 | ND | Collective, Outdoors | Dirt | No | UUW |
| 28 | ND | ND | ND | ND | ND | ND | UUW |
| 29 | ND | 295 | 2111 | Collective, Outdoors | Dirt | No | UUW |

ND: not determined. UUW: untreated underground water.
